# Nux Vomica in Intestinal Obstructions

**Published:** 1849-11

**Authors:** 


ARTICLE III.

*Nux Vomica in Intestinal Obstructions.*—It is notorious that certain obstinate cases of intestinal obstruction owe their difficulty to our ignorance of their cause; equally notorious that our practice is often empirical. In the "Tiansactions of the Medical Society of Rouliers," Dr. Oussieur has communicated some valuable information concerning the use of nux vomica in such cases. He says it produces a degree of excitement, more or less energetic, where there is deficient intestinal innervation, which often restores them to their natural action.—Assuming this—and the fact is undisputed—we cannot refuse our assent to the doctrine that the medicine may act upon the muscular fibres of the intestines as it does on the muscles generally. In support of this view, Dr. Ossieur refers to the action of nux vomica in chronic catarrh, with relaxation of the mucous membrane, to lead colic, to prolapsus ani in children, and to atonic diarrhœa. He relates two cases of obstinate constipation, which, resisting all other means, yielded at once to the nux vomica.—*Medical Times*, May 26, 1849.

